# The Influence of Solar-Simulated UV Radiation on Circulating 25(OH)D3, 24,25(OH)_2_D3 and Their Ratio in Younger and Older Adults

**DOI:** 10.3390/nu17122039

**Published:** 2025-06-18

**Authors:** Oktawia P. Borecka, Jonathan C. Y. Tang, William D. Fraser, Lesley E. Rhodes, Ann R. Webb

**Affiliations:** 1 Department of Earth and Environmental Sciences, Faculty of Science and Engineering, University of Manchester, Manchester M13 9PL, UK; 2 Photobiology Unit, Dermatology Research Centre, Salford Royal Hospital, Northern Care Alliance NHS Foundation Trust, Manchester Academic Health Science Centre, Manchester M6 8HD, UK; 3 Division of Musculoskeletal and Dermatological Sciences, School of Biological Sciences, Faculty of Biology Medicine and Health, University of Manchester, Manchester M13 9PL, UK; 4 Bioanalytical Facility, Norwich Medical School, University of East Anglia, Norwich Research Park, Norwich NR4 7TJ, UK; 5 Departments of Clinical Biochemistry and Endocrinology, Norfolk and Norwich University Hospital, Norwich NR4 7UY, UK

**Keywords:** vitamin D, skin, UV radiation, ageing, 25-VMR

## Abstract

**Background:** In addition to the well-known vitamin D metabolites 25(OH)D and 1,25(OH)_2_D, the catabolite 24,25(OH)_2_D may also reflect vitamin D status and influence biological and skeletal processes. However, the effects of UVR-induced synthesis on 24,25(OH)_2_D levels and the 25-VMR (24,25(OH)_2_D3:25(OH)D3 ratio) remain unclear. **Objectives:** We aimed to assess how a single standardised UVR dose influences the production of 25(OH)D3, 24,25(OH)_2_D3, 1,25(OH)_2_D3 and 25-VMR, with a comparison between younger and older adults being conducted to explore potential age-related differences in vitamin D metabolism. **Methods**: A total of 11 young (18–40 years; 7M, 4F) and 10 older (65–89 years; 6M, 4F) skin type I-III volunteers received a single sub-erythemal dose of solar simulated UVR (SSR) (95% UVA: 320–400 nm, 5% UVB: 290–320 nm, 1.3 standard erythemal dose) during winter time in the UK (vitamin D trough season), exposing approximately 35% of the body surface area. The Blood was assayed for 25(OH)D3, 24,25(OH)_2_D3 and 1,25(OH)_2_D3 using LC-MS/MS at baseline, 24 h and 7 days following UVR exposure. **Results:** There was a significant increase in 25(OH)D3 from baseline (44 ± 22 nmoL/L) to 24 h post-UVR (48 ± 22 nmoL/L) in the combined age group (*p* = 0.044), but no significant differences were found in 24,25(OH)_2_D3 in the combined group, or between young and older volunteers for both metabolites. 1,25(OH)_2_D3 concentrations were higher in young groups (163 ± 60 pmoL/L) than in older (105 ± 38 pmoL/L) groups at 7 days post-UVR (*p* = 0.044). The 25-VMR decreased from baseline (9 ± 3) to 24 h post-UVR (7.5 ± 2.1) in the combined group (*p* = 0.003). **Conclusions:** Our data suggest that a single sub-erythemal UVR challenge does not influence 24,25(OH)_2_D3 concentration in younger and older adults at 24 h and 7 days post-UVR and that the significant difference seen in the 25-VMR between baseline and 24 h post-UVR is due to the increase in 25(OH)D3 concentration post-UVR. This is in line with vitamin D oral supplementation studies, and indicates that low doses of UVR trigger the metabolic pathway, without affecting the catabolic pathway.

## 1. Introduction

**Figure 1:**
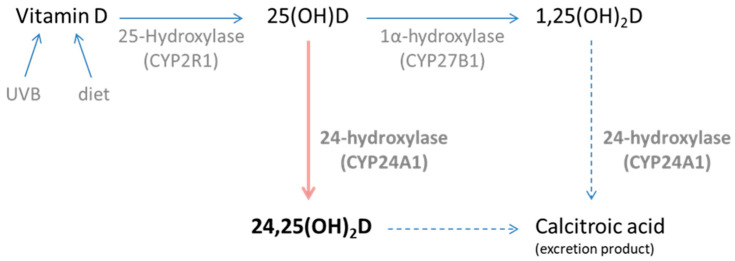
Vitamin D metabolism involving 24-hydroxylase (CYP24A1) enzyme. Vitamin D can be sourced through UVR exposure of the skin or through diet/supplementation. The 25(OH)D produced can be converted in the kidneys into 1,25(OH)2D, regulated by vitamin D status. Separately, it can also be converted into 24,25(OH)2D in various tissues expressing the vitamin D receptor. The activity of the CYP27B1 and CYP24A1 (in the excretory pathway) is tightly regulated by the concentration of 1,25(OH)_2_D, parathyroid hormone (PTH) and calcium in serum [2,3].

Vitamin D is an essential hormone for musculoskeletal health throughout human life. The cutaneous vitamin D pathway starts with skin exposure to ultraviolet B (UVB) radiation. Precursor 7-dehydrocholesterol (7DHC) is rapidly converted into pre-vitamin D3 upon irradiation, which then undergoes slower heat isomerisation to vitamin D3 (cholecalciferol) [1]. This final product of skin synthesis enters the blood attached to vitamin D binding protein (VDBP). Cholecalciferol is transported to the liver to be converted into 25-hydroxyvitamin D (25(OH)D) by 25-hydroxylase (CYP2R1) enzyme, and then to the kidney to be converted into the most biologically active form of vitamin D, calcitriol (1,25(OH)_2_D), through 1-hydroxylase. However, the vitamin D pathway involves many other compounds besides 25(OH)D and 1,25(OH)_2_D, such as 24,25-dihydroxyvitamin D (24,25(OH)_2_D) (Figure 1). The existence of an alternative vitamin D pathway involving this compound indicates that catabolism of 25(OH)D is a more complex process than traditionally assumed.

Mixed results from vitamin D supplementation trials [4,5] indicate other processes taking place in regulating vitamin D status besides the metabolites in the classic vitamin D pathway. One candidate is 24,25(OH)_2_D, the function and mechanism of which used to be considered unclear [6,7], though some animal studies suggest that it might have bone formation functions [8,9]. Moreover, there are indications that impaired renal production of 1,25(OH)_2_D has links to reduced activity of CYP24A1, which encodes the 24-hydroxylase enzyme [10]. This enzyme converts 25(OH)D into 24,25(OH)_2_D; therefore, the concentration of 24,25(OH)_2_D might also be a useful indicator of loss-of-function mutations of CYP24A1 in patients. A catabolic pathway is a sequence of biochemical reactions that degrade vitamin D metabolites into simpler compounds, ultimately leading to their excretion and regulating their biological activity. Overall, the 24,25(OH)2D3:25(OH)D3 ratio (25-VMR, vitamin D3 metabolite ratio) might be able to indicate the catabolic status of the vitamin D system [11]. In recent years, the diagnostic cut off for 24,25(OH)_2_D-replete status has been proposed as >4.2 nmoL/L in a study involving 1996 healthy subjects (mean age 23; range 18–32 years old) and 294 hospital patients (mean age 52; range 2–95) [12].

The impact of vitamin D supplementation on the relationship between 24,25(OH)_2_D and 25(OH)D has been examined. The conclusion from two independent clinical trials was that large bolus doses of vitamin D increase the activation of the catabolic pathway, leading to greater production of 24,25(OH)_2_D3, compared to smaller doses [13,14]. In these, Saleh et al. [13] supplemented 52 subjects who had 25(OH)D < 50 nmoL/L with a single 100,000 IU oral dose of vitamin D3 and 55 with a placebo, while Ketha et al. [14] supplemented 20 lactating women with either a single dose of vitamin D3 (150,000 IU) or a daily dose (5000 IU) for 28 days. Other studies also concluded that there is a positive relationship between 24,25(OH)_2_D3 and 25(OH)D3 concentration, including a study by Wagner et al. [15] where healthy young volunteers received either 28,000 IU vitamin D3 per week, fortified cheese of equivalent bioavailability (*n* = 60), or a placebo (*n* = 20) for eight weeks during winter months, and 24,25(OH)_2_D3 concentrations were measured at week two and six. The study by Tang et al. [12] found a positive, concentration-dependent relationship between serum 24,25(OH)_2_D and 25(OH)D concentrations, indicating that as levels of 25(OH)D increase, so do levels of 24,25(OH)_2_D. Further, Cashman estimated that, on average, the 24,25(OH)_2_D3 concentration in serum represented 9% of the serum 25(OH)D3 concentration in a supplementation trial with 125 ≥50-year-old volunteers [16,17].

The findings of the supplementation studies indicate a potential, direct relationship between high 24,25(OH)2D concentrations and the intake of large oral bolus doses of vitamin D. However, it is not known how the 25-VMR metabolites’ relationship and ratio may be influenced by UVR and how the former may change with age. Our study aims to compare vitamin D metabolite differences between younger and older adults, in order to explore potential age-related variations in vitamin D metabolism that could impact recommendations for UVR exposure and vitamin D supplementation across different age groups. Here, in a standardised UVR intervention study mimicking natural solar UVR exposure, we explore the ratio of metabolites in cohorts of younger and older healthy volunteers.

## 2. Materials and Methods

### 2.1. Study Cohort

This study was performed in healthy volunteers, in the Photobiology Unit, Dermatology Research Centre, University of Manchester, based at Salford Royal Hospital, NCA NHS Foundation Trust, Greater Manchester, UK. Inclusion criteria were as follows: healthy volunteers, ambulant male or female adults, and phototype I–III (white Caucasian) individuals, aged 18–40 years old or 65–89 years old. Exclusion criteria were as follows: a history of skin cancer/photosensitivity, use of a sunbed/sunbathing within 3 months, photoactive medication/bone active therapies, vitamin D doses >200 IU (5 μg)/day, and anticoagulants (including Aspirin, Clopidogrel, Warfarin) or Propranolol.

Participants, all from Greater Manchester, UK, were recruited through advertisements and the Photobiology Unit healthy volunteer database (November 2018–January 2019). They completed the study in the winter, from January to March 2019. Four potential volunteers were excluded: three younger adults did not fit the skin type criteria, while one older adult was undergoing immune-suppressant therapy.

The North West Greater Manchester West Research Ethics Committee provided ethical approval (reference 18/NW/0493). This study adhered to the Declaration of Helsinki; all volunteers gave written, informed consent.

### 2.2. Simulated Summer Sunlight Exposures

Volunteers were given a single sub-sunburn dose of solar-simulated UVR, at a 1.3 standard erythemal dose (SED), equivalent to about 15 min of midday summer exposure in the UK. This was performed during January-March, when ambient UVB is insufficient to produce appreciable vitamin D3 in the skin at the study site latitude (53.5° N) and volunteers would be at the nadir of their annual cycle in vitamin D status. This dose took 6 min 20 s to administer.

A whole-body Philips HB588 Sunstudio irradiation cabinet (Philips, Eindhoven, The Netherlands) was re-purposed and fitted with Arimed B lamps (Cosmedico GmbH, Stuttgart, Germany), providing a UVR emission close to that provided by UK summer sunlight (emission 290–400 nm; 95% UVA: 320–400 nm, 5% UVB: 290–320 nm). The cabinet emission was characterised using a DTM300 spectroradiometer (Bentham, Reading, UK) at the beginning of the study.

While exposed to radiation and lying prone, subjects wore standardised clothing (shorts and T-shirt) to expose their hands, forearms, face, and lower legs, comprising an approximately 35% body surface area. There was a 10 cm × 10 cm cut-out area on one side of the shorts to expose an upper-buttock area (UVR exposed site), and the other buttock was covered with UVR-opaque material (unexposed site). Volunteers were also supplied with and asked to wear protective eye goggles.

### 2.3. Parathyroid Hormone and Serum Biochemistry

Blood samples were taken on three occasions during the study: the first visit (baseline assessments), 24 h post-UVR and 7 days post-UVR. Peripheral blood (approx. 20 mL) was separated by centrifugation, and serum samples were stored at −80 °C prior to analysis. Routine biochemistry, including renal function and parathyroid hormone (PTH), was analysed at Salford Royal Hospital, Greater Manchester, UK. Analysis of PTH was carried out using a 2-site sandwich immunoassay using direct chemiluminometric technology (Siemens Centaur XP Intact PTH assay), and routine biochemistry was analysed by the Siemens ADVIA assay.

### 2.4. Circulating Vitamin D Derivatives Analysis

Serum analysis of circulating vitamin D metabolites, including 25(OH)D3, 24,25(OH)_2_D3, and 1,25(OH)_2_D3, was performed via liquid chromatography tandem mass spectrometry (LC-MS/MS) at the University of East Anglia (UEA) Bioanalytical Facility (BAF), Norwich, UK, under good clinical and laboratory practice conditions. Analysis of serum 25(OH)D3 and 24,25(OH)_2_D3 was conducted using a Micromass Quattro Ultima Pt LC-MS/MS (Waters Corp., Milford, MA, USA), as described previously [12,18]. The measurement ranges of the assays were 0.1–200 nmol/L for 25(OH)D3 and 0–25 nmol/L for 24,25(OH)_2_D3, calibrated using standard reference material SRM972a from the National Institute of Science and Technology (NIST). The mean coefficients of variation (CVs) for intra-assay imprecision across the measuring range of the assays were between 4.9 and 9.6%. The 25(OH)D assays showed a <6% accuracy bias against the Vitamin D External Quality Assessment Scheme (DEQAS) reference LC-MS/MS provided by the Centres for Disease Control and Prevention (CDC, Atlanta, GA, USA) and met the certification performance standards set by DEQAS throughout the time the analyses were performed. Deficiency of vitamin D was defined as a 25(OH)D concentration below 25 nmol/L (10 ng/mL), and sufficiency was defined as a concentration of 50 nmol/L (20 ng/mL) and above [19,20,21]. The 25-VMR was expressed in a percentage calculated by 24,25(OH)_2_D3/ 25(OH)D3 × 100.

1,25(OH)_2_D3 was analysed by a Waters Xevo TQ-XS LC-MS/MS system (Waters Corp., Milford, MA, USA) as described in [22]. The assays showed a linearity between 0 and 900 pmol/L. The inter/intra-assay CVs were between 2.0 and 8.5% across the assay range, with the lower limit of quantification (LLoQ) at 10 pmol/L. During the period of analysis, the assay showed <3.3% accuracy bias against the DEQAS LC-MS/MS method’s group mean.

### 2.5. Statistical Analyses

The sample size was limited by the number of adults willing to participate in studies that required multiple skin and blood samples. The sample size was estimated using confidence interval methodology, based on the primary outcome of detecting changes in 7DHC concentration in the skin, as described in a previous publication [23]. 25(OH)D3, 24,25(OH)_2_D3 and 1,25(OH)_2_D3 results were assessed for normality using the Shapiro–Wilk test and QQ plots. The 25-VMR data were transformed using the equation y = ln(y), and all further analyses were carried out with transformed data in the same way as for other datasets. All sets of data were analysed using mixed-effects analysis and a multiple comparisons (Sidak correction) test using GraphPad Prism statistical software (version 8.4.3, 10 June 2020). Post hoc comparisons between time points were performed regardless of the overall ANOVA result, in order to explore potential time- and age-dependent effects.

## 3. Results

A total of 25 volunteers were recruited, including 14 and 11 in the younger and older age groups, respectively. One participant from the 18–40-year-old cohort withdrew from participating, and one of the participants in the 65–89-year-old group was not able to attend the 24 h post-UVR exposure. One of the younger volunteers had to be excluded as she failed to refrain from a sunny holiday, and another was excluded because their PTH level was abnormally high. One participant in the older group was excluded due to prior undisclosed vitamin D supplementation. Complete datasets were available for 8 younger volunteers and 9 older volunteers, with partial datasets available for a further 3 younger volunteers and 1 older volunteer. All available data were used in the analysis. Table 1 shows volunteer demographics and daily vitamin D intake, as described in a previous publication [24] and Table 2 shows their baseline biochemistry data.

### 3.1. 25(OH)D3

**Figure 2:**
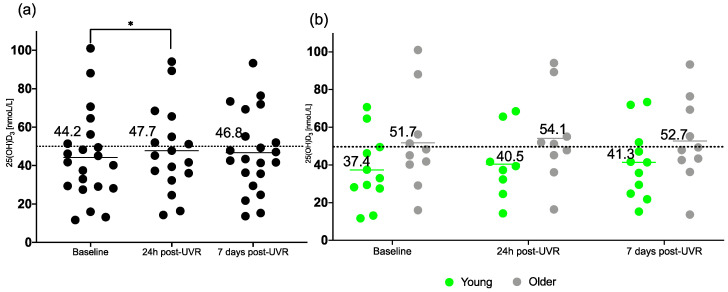
Concentration of serum total 25(OH)D3 in (**a**) all volunteers (*n* = 21/17/21), and in (**b**) the young (*n* = 11/8/11) and older (*n* = 10/9/10) groups, during the course of the study at baseline, 24 h post-UVR and 7 days post-UVR. * indicates a significant *p* value of <0.05. The dotted line represents the sufficiency cut off (50 nmol/L). Horizontal lines indicate the means.

Serum 25(OH)D is the standard measure of vitamin D status. Here, we report specifically on 25(OH)D3, which is obtained from production in the skin, as well as orally. Figure 2 shows the time course of 25(OH)D3 for all volunteers, i.e., both younger and older age groups. The mean baseline, 24 h and 7-day post-UVR concentration of 25(OH)D3 was lower in the younger age group than in the older group, contrary to traditional expectations [25,26]. The mean 25(OH)D3 concentration in the older group was above the sufficiency level (>50 nmoL/L) at all time points. In contrast, younger volunteers’ 25(OH)D3 mean concentration did not reach sufficiency during the study.

Mixed-effects analysis showed no significant difference in concentrations of 25(OH)D3 in the groups combined at different timepoints (F (0.1800, 3.240) = 1.416; *p* = 0.16); however, multiple comparison testing showed a significant increase in 25(OH)D3 between the baseline and 24 h post-UVR (*p* = 0.044). No significant difference was found in the age, time and interaction of time and age for concentrations of 25(OH)D3 in young vs. older groups. 25(OH)D3 means and standard deviations for all groups are provided in Table 3.

### 3.2. 24,25(OH)_2_D3

**Figure 3:**
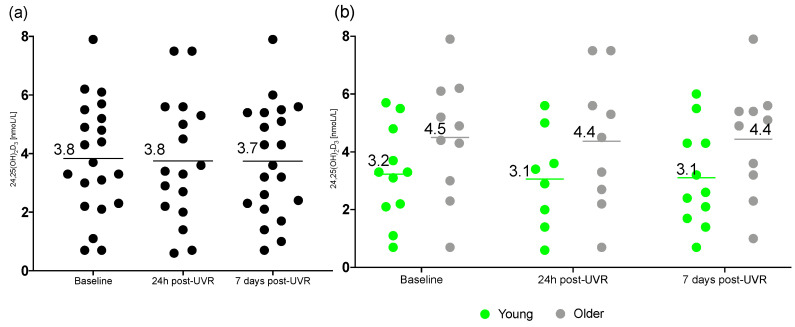
Concentration of serum 24,25(OH)_2_D3 in (**a**) all participants (*n* = 21/17/21), and in (**b**) the young (*n* = 11/8/11) and older (*n* = 10/9/10) group, at baseline, 24 h post-UVR and 7 days post-UVR. Horizontal lines indicate means.

The effects of UVR on 24,25(OH)2D3 concentration are quantified here. The mean 24,25(OH)2D3 concentration in the older group of volunteers was above the diagnostic cut off for replete status (>4.2 nmoL/L) [12] at all time points, whereas younger volunteers’ total 24,25(OH)2D3 mean concentration did not reach sufficiency during the trial (Figure 3).

Mixed-effects analysis showed no significant difference in the concentrations of 24,25(OH)_2_D3 in groups combined at different time points (F (1.084, 19.51) = 0.4888; *p* = 0.51) and no differences were found in multiple comparison testing. Also, no significant difference was found in the age, time and interaction of time and age for total concentrations of 24,25(OH)_2_D in young vs. older groups. 24,25(OH)_2_D3 means and standard deviations for all groups are provided in Table 3.

### 3.3. Vitamin D3 Metabolite Ratio (25-VMR)

**Figure 4:**
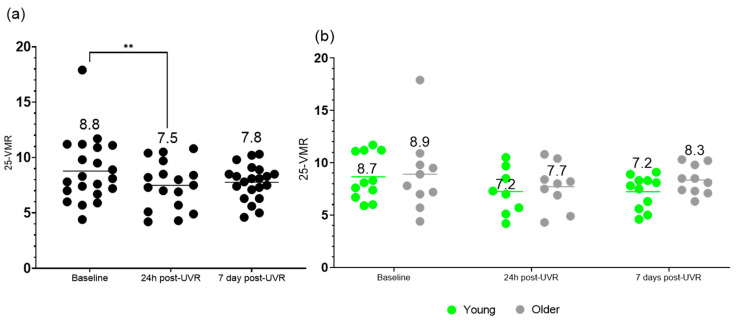
Percentage ratio of serum 24,25(OH)_2_D3 to 25(OH)D3 (25-VMR) in (**a**) all participants (*n* = 21/17/21), and in (**b**) younger (*n* = 11/8/11) and older (*n* = 10/9/10) groups, at baseline, 24 h post-UVR and 7 days post-UVR. All statistical analyses were performed on transformed data (y = ln(y)). ** indicates a *p* value of <0.01. Horizontal lines indicate means.

Here, the effects of a single dose of UVR on the 25-VMR is presented (Figure 4).

Mixed-effects analysis showed no significant difference in 25-VMR in groups combined at different time points (F (1.884, 33.92) = 2.963; *p* = 0.068); however, a multiple comparison test indicated a significant difference in 25-VMR between baseline and 24 h post-UVR in the combined groups (*p* = 0.003). No significant difference was found for age, time, or the interaction of time and age in young versus older volunteers.

### 3.4. 1,25(OH)_2_D3

**Figure 5:**
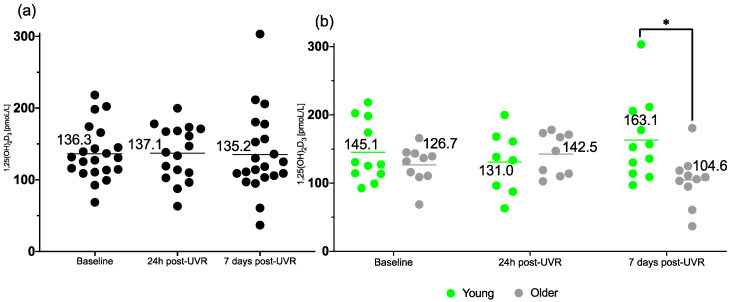
Concentration of serum 1,25(OH)_2_D3 of (**a**) all volunteers (*n* = 21/17/21) and in the (**b**) younger (*n* = 11/8/11) and older (*n* = 10/9/10) groups at baseline, 24h post-UVR and 7 days post-UVR. * indicates a significant *p* value of <0.05. Horizontal lines indicate means.

The production of 1,25(OH)_2_D is tightly regulated [27], with PTH playing a crucial role in stimuli for 1,25(OH)_2_D synthesis in the kidney [28]. Here, we present 1,25(OH)_2_D3, which is produced from 25(OH)D3 (Figure 5).

Mixed-effects analysis showed no significant difference in the concentrations of 1,25(OH)_2_D3 in groups combined at different time points (F (1.742, 31.36) = 0.01324; *p* = 0.98). Mixed-effects analysis of the young vs. older groups showed a significant difference in the interaction of time and age (F (2, 34) = 5.928; *p*= 0.006), and in multiple comparisons, there was a significant difference in 1,25(OH)_2_D3 concentrations between the young and older groups 7 days post-UVR (*p* = 0.044).

### 3.5. Molar Ratio of 1,25(OH)_2_D3 to 24,25(OH)_2_D3 VMR

**Figure 6:**
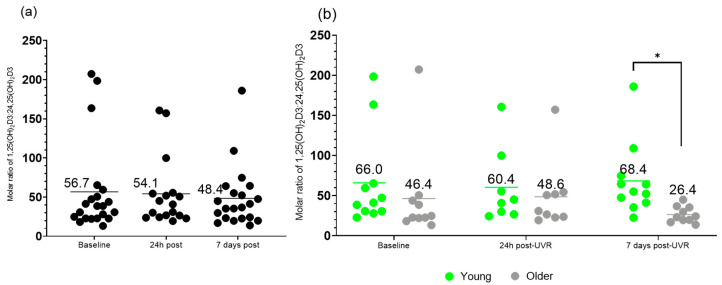
Molar ratio of serum 1,25(OH)_2_D3 to 24,25(OH)_2_D3 VMR in (**a**) all participants (*n* = 21/17/21), and in (**b**) the young (*n* = 11/8/11) and older (*n* = 10/9/10) groups, at baseline, 24 h post-UVR and 7 days post-UVR. All statistical analyses were performed on transformed data (y = 1/y). * indicates *p* value of <0.05. Horizontal lines indicate means.

Here, the effects of a single dose of UVR on the molar ratio of 1,25(OH)_2_D3 to 24,25(OH)_2_D3 VMR is presented (Figure 6).

Mixed-effects analysis showed no significant difference in the ratio of 1,25(OH)_2_D3 to 24,25(OH)_2_D3 VMR in groups combined at different time points (F (1.869, 33.64) = 0.3577; *p* = 0.69). Mixed-effects analysis of young vs. older groups showed a significant difference in the interaction of time and age (F (2, 34) = 7.501; *p*= 0.002) and age (F (1, 19) = 5.784; *p*= 0.03). In multiple comparisons, there was a significant difference in the 1,25(OH)_2_D3 to 24,25(OH)_2_D3 VMR between young and older groups 7 days post-UVR (*p* = 0.004).

## 4. Discussion

In this study, we investigated whether UVR exposure of the skin influences 24,25(OH)_2_D3 concentration and the 25-VMR. We recruited healthy volunteers matched by skin type and performed all procedures in a standardised fashion. The study was performed in winter months in the UK, when ambient UVB is insufficient to produce any biologically appreciable vitamin D3. Volunteers were also excluded if they had been on a sunny holiday in the prior 3 months or were taking >200 IU/day of vitamin D supplements. The UV irradiation protocol was designed to mimic natural sunlight exposure in humans, using a UVR comprising 95% UVA and 5% UVB (similar to ambient UVR in summer), with ~35% skin surface area exposure (as when wearing summer clothes), and a low sub-erythemal UVR dose was applied that is believed to be most efficient for vitamin D3 synthesis [29,30].

The data collected in our study show that there was a significant increase in 25(OH)D3 from baseline to 24 h post-UVR in the age groups combined (*p* = 0.044), accompanied by an increase in 25-VMR (*p* = 0.003). This suggests that the significant difference in the ratio between baseline and 24 h post-UVR is due to the increase in 25(OH)D3, as we found that 24,25(OH)_2_D3 concentrations did not change significantly during the study. The concentrations of 1,25(OH)_2_D3 were not significantly different between the age groups, apart from at 7 days post-UVR, when the mean concentration in the older group was significantly lower than that of the younger group. This may be an anticipated outcome considering the decreased kidney filtration rate, which is known to occur with age [31], and the lack of a direct relationship between 25(OH)D and 1,25(OH)_2_D [18]. Our results also show the lack of a direct relationship between UVR exposure and the 1,25(OH)_2_D3 level. No significant differences were found in 25(OH)D3, 24,25(OH)_2_D3, or 25-VMR at different timepoints when the younger vs. older group were compared, although this could be explained by smaller sample sizes compared to when the volunteers’ data are combined.

What we have observed in our study is that a small dose of UVR did not affect 24,25(OH)_2_D3; however, it allowed for a small yet significant increase in 25(OH)D3 24 h post-UVR. This is consistent with the findings of oral supplementation studies where baseline 25(OH)D3 and 24,25(OH)_2_D3 were measured and compared with the concentrations after supplementation. A trial comparing a daily dose of vitamin D (5000 IU) to a large bolus dose (150,000 IU) showed that high-dose vitamin D led to a greater production of 24,25(OH)_2_D3 than a lower daily dose [14]. It was hypothesised that this is due to the induction of CYP24A1 with the increased conversion of 25(OH)D3 into 24,25(OH)_2_D3, thus favouring the catabolic pathway. A study comparing the effect of a placebo to a large single 100,000 IU oral dose of vitamin D3 in participants with 25(OH)D3 concentrations below 50 nmoL/L showed that the 25-VMR remained unchanged on the placebo, but significantly increased in the group supplemented with the bolus dose [13]. Overall, the literature supports the view that smaller daily doses of oral vitamin D are more effective than high-dose oral bolus in increasing 25(OH)D3 production, while avoiding an increase in the catabolic pathway, and our report of impact of a low UVR dose appears to be in agreement with this. It gives further support to the sun exposure messages of ‘brief exposures’, which are based upon the efficacy of the earlier portion of the vitamin D pathway, i.e., conversion of 7DHC into pre-vitamin D3 [32], but also appear to support efficiency in the metabolite pathways. UV doses below the threshold for erythema (i.e., <1 Minimal Erythema Dose) have been shown to effectively stimulate vitamin D synthesis in the skin, particularly when repeated exposures are applied over time [33]. Such sub-erythemal regimens are considered safe for most individuals, provided that factors such as skin phototype, baseline vitamin D status, and body surface area exposed are taken into account. Although the observed ~3.5 nmol/L increase in serum 25(OH)D3 may appear modest, comparable longitudinal data—such as an adjusted 2.7 nmol/L rise in 25(OH)D3 over 10 years—have been associated with significant decreases in PTH [34]. This indicates that even small improvements in vitamin D status may hold biological significance, particularly in the context of long-term calcium regulation and skeletal health.

Furthermore, our data also suggests that the concentrations of 25(OH)D3 and 24,25(OH)_2_D3 might be directly related. While our clinical study was small, we found that the mean 25(OH)D3 in the older group of volunteers was above the 50 nmol/L cut off for sufficiency, and the mean concentration of 24,25(OH)_2_D3 was below the >4.2 nmol/L threshold previously proposed to reflect vitamin D replete status [12]. This relationship was also apparent in the younger group, although mean concentrations of 25(OH)D3 and 24,25(OH)_2_D3 were both below the respective cut offs, further suggesting a link between these two derivatives, as described in larger sample oral supplementation studies [12,13,14]. In a clinical trial of 160 healthy, young adults who received 8 weekly doses of 28,000 IU, there was a strong correlation between 24,25(OH)_2_D3 and 25(OH)D3, suggesting that the catabolism of 25(OH)D3 into 24,25(OH)D3 increases with increasing concentrations of 25(OH)D3 [15]. A further large trial investigated this relationship while supplementing volunteers with a placebo or a daily 800 IU dose of vitamin D3. The supplementation significantly increased both serum 25(OH)D3 and 24,25(OH)_2_D3 concentrations, but also 25-VMR, hence signifying the increased catabolic activity of CYP24A1 [16,17].

Consequently, 24,25(OH)_2_D, in addition to 25(OH)D, may be useful as a predictor of the vitamin D status of a person [11,17,34,35,36]. 24,25(OH)_2_D values below 4.2 nmoL/L suggest that the concentration of 25(OH)D might also be below <50 nmol/L, which is commonly used as the threshold for vitamin D deficiency or insufficiency [19,20,21], and that metabolism is focused on the production of 1,25(OH)_2_D rather than 24,25(OH)_2_D [12]. Parallel 24,25(OH)_2_D and 25(OH)D measurement also permits the assessment of the activity of CYP24A1 (24-hydroxylase) enzyme, which can reveal other potential health problems or genetic mutations in vitamin D catabolic enzymes [35,37,38,39,40]. LC-MS/MS methods not only provide higher accuracy than immunoassay methods [41,42], but also allow for the simultaneous measurement of 24,25(OH)_2_D and 25(OH)D, which can be a way forward in clinical testing for vitamin D status and can be applicable via its attainment by both oral and UVR sources.

A notable reduction in serum 1,25(OH)_2_D 3 concentrations was observed in the older group at 7 days post-UVR, with an approximate 30% decrease from those at 24 h post-UVR. Although this reduction did not reach statistical significance—likely due to the limited sample size—it remains a physiologically relevant finding and warrants further investigation in larger cohorts. Given the tightly regulated nature of 1,25(OH)_2_D homeostasis, this decline may reflect a compensatory response to the net increase in 25(OH)D3 following UVR exposure, particularly as 24,25(OH)_2_D3 levels remained unchanged. Such a mechanism could serve to mitigate calcium release, thereby potentially reducing bone resorption over time and contributing to skeletal health.

However, a key limitation of this study is the relatively small sample size, which was influenced by the invasive nature of the protocol, including repeated skin biopsies. While it was ethically and practically necessary, the small cohort size reduced the statistical power and generalisability of the findings. This should be taken into account when interpreting non-significant results and apparent similarities in responses between age groups, as these findings may reflect insufficient power rather than true equivalence. Further studies with larger cohorts, the inclusion of a “sham” exposure group (e.g., no UVR) to strengthen causal inference, and additional blood sampling timepoints are needed to expand upon these observations.

## 5. Conclusions

Overall, the data collected in our study suggest that low-dose UVR may influence 24,25(OH)_2_D3 and 25(OH)D3 and their ratio in a similar way to lower-dose oral supplementation. Thus, sub-erythemal dosing with UVR appears to trigger the metabolic pathway, without affecting the catabolic pathway. Further, this finding might support the sun exposure message of “brief exposures” or ‘little and often’ [31]. We have not observed a difference in response to UVR between the younger and older group, suggesting that younger and older adults might respond similarly to UVR in terms of 25(OH)D3 and 24,25(OH)_2_D3 production. However, this study was limited by the small sample size of the individual groups. Larger trials assessing the effects of oral supplementation and UVR on younger and older adults may be warranted, in addition to further human in vivo studies examining the impact of repeated UVR dosing.

## Figures and Tables

**Table 1:** Volunteer demographics.

	Younger	Older
Participants, *n*	11 (after 1 withdrew, 2 excluded)	10 (after 1 excluded)
Sex: male, female (*n*)	7, 4	6, 4
Age (years) ± SD	29.5 ± 6.3	70.2 ± 3.8
BMI (kg/m^2^) ± SD	27.1 ± 4.8	26.8 ± 5.6
Skin type I, II, III (*n*)	3, 2, 6	1, 2, 7
Oral vitamin D intake (IU/day)	96 ± 10	160 ± 22

Note: Mean values, unless otherwise stated.

**Table 2:** Volunteer baseline biochemistry data.

	Younger					Older					
	Minimum	Lower Quartile	Median	Upper Quartile	Maximum	Minimum	Lower Quartile	Median	Upper Quartile	Maximum	Normal Range
Parathyroid hormone (pmoL/L)	2.4	3.2	4.1	4.5	8.2	2.5	3.5	4.3	5.4	6.6	1.5–7.6
eGFR	77	93	110	114	127	59	66	74	85	131	>60
Sodium (mmoL/L)	137	138	139	140	144	137	138	139	140	142	133–146
Potassium (mmoL/L)	3.9	4.2	4.2	4.4	4.6	3.6	4.1	4.2	4.5	4.7	3.5–5.3
Urea (mmoL/L)	3.4	4.0	4.4	5.1	7.9	4.5	5.4	5.9	6.4	7.0	2.5–7.8
Creatinine (μmoL/L)	49	61	73	80	95	52	76	81	84	100	62–115 (m) 44–97 (f)
Adjusted Calcium (mmoL/L)	2.3	2.3	2.4	2.4	2.5	2.3	2.3	2.4	2.4	2.6	2.2–2.6
Phosphate (mmoL/L)	0.95	1.04	1.12	1.27	1.39	0.97	1.15	1.17	1.21	1.5	0.8–1.5
Albumin (g/L)	46	47	47	48	51	43	45	45	47	48	35–50

**Table 3:** Summary of means and standard deviations of vitamin D metabolites and their ratio for all groups.

	Groups Combined	Younger	Older
**25(OH)D3** (nmoL/L)			
Baseline	44.2 ± 22.9 *	37.4 ± 18.9	51.7 ± 25.5
24 h post-UVR	47.7 ± 22.3 *	40.5 ± 18.6	54.1 ± 24.3
7 d post-UVR	46.8 ± 21.0	41.3 ± 19.0	52.7 ± 22.4
**24,25(OH)_2_D3** (nmoL/L)			
Baseline	3.8 ± 1.9 **	3.2 ± 1.7	4.5 ± 2.1
24 h post-UVR	3.8 ± 2.1 **	3.1 ± 1.7	4.4 ± 2.3
7 d post-UVR	3.7 ± 1.9	3.1 ± 1.7	4.4 ± 2.0
Vitamin D Metabolite Ratio [25-VMR = 100 * (24,25(OH)_2_D3/25(OH)D3)]			
Baseline	8.8 ± 2.9	8.7 ± 2.2	8.9 ± 3.7
24 h post-UVR	7.5 ± 2.1	7.2 ± 2.2	7.7 ± 2.0
7 d post-UVR	7.8 ± 1.0	7.2 ± 1.6	8.3 ± 1.4
**1,25(OH)_2_D3** (pmoL/L)			
Baseline	136.3 ± 37.6	145.1 ± 44.7	126.7 ± 26.9
24 h post-UVR	137.1 ± 37.9	131.0 ± 45.9	142.5 ± 30.9
7 d post-UVR	135.2 ± 57.8	163.1 ± 59.7 *	104.6 ± 38.1 *
**Molar ratio of 1,25(OH)_2_D3 to 24,25(OH)_2_D3**			
Baseline	56.7 ± 57.7	66.0 ± 58.8	46.4 ± 57.8
24 h post-UVR	54.1 ± 44.0	60.4 ± 47.2	48.6 ± 40.5
7 d post-UVR	48.4 ± 39.0	68.4 ± 45.1	26.4 ± 10.0

* indicates a significant *p* value of <0.05; ** indicates a *p* value of <0.01.

## Data Availability

Data available on request from the corresponding authors. The data are not publicly available due to ethical and privacy restrictions, as they originate from a clinical study involving human participants.
